# Prospective Observational Case Series in Infertile Women with Overweight or Obesity Treated with a Very-Low Calorie Ketogenic Diet (VLCKD) Prior to an In Vitro Fertilization (IVF) Treatment

**DOI:** 10.3390/nu17182930

**Published:** 2025-09-11

**Authors:** Maíra Casalechi, Alessandra Piontini, Annaelisa Nicolosi, Francesca Bergomas, Filomena Napolitano, Stefano Turolo, Marco Reschini, Alessandra Riccaboni, Roberta Bellinghieri, Edgardo Somigliana, Luisella Vigna

**Affiliations:** 1 Infertility Unit, Fondazione IRCCS Ca’ Granda Ospedale Maggiore Policlinico, 20122 Milan, Italy; maira.casalechi@policlinico.mi.it (M.C.); annaelisa.nicolosi@policlinico.mi.it (A.N.); marco.reschini@policlinico.mi.it (M.R.); alessandra.riccaboni@policlinico.mi.it (A.R.); roberta.bellinghieri@policlinico.mi.it (R.B.); edgardo.somigliana@policlinico.mi.it (E.S.); 2 Occupational Health Unit, Obesity and Work Center, Fondazione IRCCS Ca’ Granda Ospedale Maggiore Policlinico, 20122 Milan, Italy; alessandra.piontini@policlinico.mi.it (A.P.); francesca.bergomas@unimi.it (F.B.); 3 Laboratory of Clinical Chemistry and Microbiology, Fondazione IRCCS Ca’ Granda Ospedale Maggiore Policlinico, 20122 Milan, Italy; filomena.napolitano@policlinico.mi.it; 4 Nephrology Dialysis and Pediatric Transplantation Unit, Fondazione IRCCS Ca’ Granda Ospedale Maggiore Policlinico, 20122 Milan, Italy; stefano.turolo@policlinico.mi.it; 5 Department of Clinical Sciences and Community Health, Università Degli Studi di Milano, 20122 Milan, Italy

**Keywords:** ketogenic diet, infertility, weight loss, very low-calorie ketogenic diet, obesity, ART, IVF

## Abstract

**Background:** Elevated BMI in women is linked to metabolic and endocrine imbalances that impair fertility and increase pregnancy risks. While >10% weight loss before an Assisted reproductive technology (ART) treatment may improve outcomes, sustained results through conventional diets are challenging. A very-low calorie ketogenic diet (VLCKD) promotes rapid fat loss while preserving lean mass and may offer long-term benefits. This study evaluated the efficacy (≥10% weight loss without lean mass reduction), adherence, metabolic effects, and pregnancy outcomes of a meal replacement VLCKD in women with overweight or obesity scheduled for ART. **Methods:** This monocentric, prospective case-series was conducted at the Obesity and Work Center, Fondazione IRCCS Ca’ Granda Ospedale Maggiore Policlinico, Milan (September 2019–September 2023). Eligible women underwent a three-phase dietary program: a 3-month VLCKD (<800 kcal/day), a 6-month transition with gradual carbohydrate reintroduction, and a Mediterranean-style maintenance diet. Participants were monitored for safety, body composition, adherence, and biochemical changes. **Results:** Of 52 women enrolled, 40 initiated the VLCKD; 27 (68%) achieved ≥10% weight loss while preserving lean mass. Eleven conceived naturally during or after the diet; 22 underwent ART, with 12 additional pregnancies. This corresponds to a 58% pregnancy rate among those who began the VLCKD. Significant improvements were observed in body mass index (BMI), fat mass, waist circumference, glucose metabolism, lipid profile, and liver function. No adverse events were reported. **Conclusions:** A meal replacement VLCKD protocol is feasible, well-tolerated, and associated with significant improvements in weight, especially in body composition, metabolic health, and potentially outcomes in women with overweight or obesity awaiting ART.

## 1. Introduction

Obesity and overweight are increasingly prevalent global health issues, closely associated with a range of complications, including reproductive dysfunction. In women, elevated body mass index (BMI) is linked to metabolic and endocrine imbalances, such as insulin resistance and altered hormonal profiles, that can negatively impact oocyte quality, embryo development, and implantation success [1,2,3]. Excess maternal adiposity may also increase inflammation and oxidative stress, raising the risk of pregnancy complications like miscarriage and preeclampsia [4,5].

The prevalence of infertility, defined by the WHO as the inability to conceive after 12 months of regular and unprotected intercourse, is increased in women with excessive body weight. Meta-analysis studies have found that female obesity negatively affects live birth rates, even in women undergoing assisted reproductive technologies (ART) [6,7]. 

While a systematic review conducted by Jeong et al. (2024) [8] concluded that programs of pre-in vitro fertilization (IVF) weight loss does not consistently improve live birth rates, other studies suggest that losing more than 10% of body weight may reduce gonadotropin requirements and improve pregnancy and live birth outcomes, even in women who remain above the “normal” BMI threshold [9]. In light of these findings, experts recommend weight loss as a preparatory step for fertility treatment in patients with obesity [5,7]. Furthermore, it is well known that obstetric outcomes are negatively impacted by BMI [4].

However, sustained weight loss via conventional diets remains challenging, even among women motivated by the desire to conceive. Time to reach target weight loss can be a major barrier, often leading to dropout or rebound weight gain—the so-called yo-yo effect [10]. Thus, efficient and sustainable interventions to improve reproductive outcomes in this population have been explored for a while [11,12]. In this context, a lifestyle intervention including simple adherence strategies tailored as preconception approaches seems ideal [13]. A very-low calorie ketogenic diet (VLCKD) has gained attention as a promising alternative. These diets drastically restrict carbohydrates while maintaining moderate protein and fat intake, inducing a state of nutritional ketosis. This promotes rapid fat loss while preserving lean body mass, which is crucial in obesity treatment [14,15], and particularly appealing for women over 38 years undergoing fertility treatments. It has been indicated that for women above this age, weight loss should occur in shorter timeframes to potentially avoid the hindering effect of advancing female age on cumulative live birth rates [16]. 

VLCKDs have also shown potential for long-term weight maintenance [17,18,19]. In the only randomized trial focusing on infertile patients scheduled for IVF, a liquid low-calorie diet did not increase the overall chance of live birth but allowed significantly more natural conceptions and achieved higher adherence than conventional low-calorie diets [20]. Yet, most studies focus on metabolic outcomes, and data specific to infertility remain limited [21]. This prospective study aimed to evaluate the success (defined as ≥10% weight loss without lean mass reduction), adherence and short-term metabolic effects of a VLCKD in female patients with overweight or obesity scheduled for ART. The primary outcome was the proportion of patients achieving a clinically significant weight reduction (≥10% of baseline body weight with preserved lean body mass) after the first phase of the diet. Secondary outcomes included changes in anthropometric, body composition, and key metabolic and biochemical parameters, as well as the frequency of natural pregnancies during the waiting period for IVF and ART-related pregnancies within an 18-month follow-up.

## 2. Materials and Methods

### 2.1. Study Design and Ethical Approval

The study was performed as a monocentric, multidisciplinary, prospective case-series study conducted at the Obesity and Work Center, of the Occupational Medicine Unit, Fondazione IRCCS Ca’ Granda Ospedale Maggiore Policlinico, Milan, Italy, between September 2019 and September 2023. 

The study was conducted in accordance with the Declaration of Helsinki and its subsequent amendments [22]. Ethical approval was obtained from the local Ethics Committee (Comitato Etico Milano Area 2; approval number: 495_2019 on 12 June 2019). All participants received detailed written and verbal information about the study’s purpose, procedures, and potential risks and benefits. Written informed consent was obtained prior to enrollment. Participation was voluntary, and individuals could withdraw at any time without penalty. If a participant became pregnant or initiated IVF during the dietary intervention period, this was recorded, and the participant was withdrawn from further procedures in accordance with the protocol.

### 2.2. Study Population

Access to IVF at Milan’s public hospital’s Infertility Unit typically involves a minimum five-month waiting period following the initial consultation. During this interval, eligible women were identified and invited to participate. Inclusion criteria were as follows: 1. age 18–43 years; 2. BMI > 25 kg/m^2^; and 3. a clinical diagnosis of infertility requiring treatment with ART. Anovulatory infertility was an indication to ART if ovarian stimulation aimed at natural conception failed. Eligibility for VLCKD was based on national guidelines, specifically the Italian Standards for the Treatment of Obesity (Italian Society for the Study of Obesity and the Italian Association of Dietetics and Clinical Nutrition, 2016–2017) and the Italian Society of Endocrinology guidelines [23]. Exclusion criteria included 1. contraindications to pregnancy, infertility treatment, or VLCKD; 2. chronic kidney disease, liver dysfunction, or type 1 diabetes; 3. active eating disorders, uncontrolled psychiatric conditions, or substance abuse; 4. medications affecting ketosis or weight regulation; and 5. severe systemic diseases, including cardiovascular disorders associated with infertility or obesity. According to the European Association for the Study of Obesity’s recommendations, inclusive and respectful language was used throughout the study to reduce bias associated with the term “obesity” and to help prevent the stigma that can arise from labeling patients by their condition [24].

### 2.3. Study Visits and Nutritional Protocol

#### 2.3.1. Recruitment Phase

Eligible women were screened based on medical history, physical examination, and existing lab results. Those meeting inclusion criteria and consenting to participate were scheduled for a baseline visit (T0) at the Obesity and Work Center of Occupational Medicine Unit. Participants were followed through three additional visits (T1–T3). Diet tolerance and safety were monitored at each visit. Significant adverse events which turned up during the diet led to shared decision-making regarding withdrawal from the study.

#### 2.3.2. Baseline Visit (T0)

At T0, participants met with a study doctor and a trained dietitian for anthropometry, body composition and complete nutritional assessment and received instructions on the VLCKD protocol. A commercial weight loss VLCKD program (Dieta ISOMED srl, Genola, Italy) was used; This program offers protein preparations with high biological value (Supplementary Table S1) in addition with natural foods. Each patient was given the option to choose among the meal replacement products and ordered their own kit. A dedicated email address was provided for adverse event reporting.

#### 2.3.3. VLCKD Phase (T0–T1)

The VLCKD provided <800 kcal/day, with 20–30 g/day of carbohydrates. Protein intake was set at 1.2 g/kg of ideal body weight, calculated based on the weight corresponding to a normal BMI of 23. The regimen included four meal replacements per day (200–267 kcal each), with macronutrient distribution of 35% protein, 52% fat (12% saturated fat), and 13% carbohydrates, including 12 g of fiber. A standardized supplementation protocol was prescribed, consisting of a multivitamin complex (IsoCompletPlus, Isomed, Genola, Italy; code: ICOM) and omega-3 fatty acids (Isomega3, Isomed, Italy; code: 210). Participants were instructed to consume a minimum of 2 L of water daily. The VLCKD phase was maintained for a duration of 3 months. A detailed overview of the dietary intervention is presented in Table 1, while an example of the VLCKD replacement meal menu is detailed in Supplementary Table S2.

#### 2.3.4. Transition Phase (T1–T2)

During the 6-month transition, individualized plans were introduced. Caloric intake increased gradually to up to 1350 kcal/day. Carbohydrates were reintroduced progressively, beginning with low-glycemic-index options, and followed by those with a moderate-glycemic-index. Macronutrient distribution during this phase was adjusted to approximately 25% protein, 43% fat (13% saturated fat), and 32% carbohydrates (19 g fiber).

#### 2.3.5. Maintenance Phase (T2–T3)

The final phase emphasized dietary stability to support a long-term dietary adherence through a Mediterranean-style pattern, with a daily caloric intake of approximately 1450 kcal. Diet in this phase was composed of 22% protein, 30% fat (including 11% saturated fat), and 48% carbohydrates (28 g of fiber).

### 2.4. Anthropometric and Body Composition Assessments

Anthropometric parameters and body composition were assessed at T0, T1 (3 months), T2 (6 months), and T3 (12 months). Weight was measured to the nearest 0.1 Kg using a calibrated Seca 201 scale, while height was measured to the nearest 0.1 cm using a stadiometer. BMI was calculated as weight in kilograms divided by height in meters squared (kg/m^2^) and categorized per World Health Organization (WHO) standards. Waist circumference (WCirc) was measured at the midpoint between the rib cage and iliac crest, using a non-stretchable SECA 201 measuring tape. 

Body composition was assessed using bioelectrical impedance analysis (BIA; Bia-Dex Mascaretti, Italy). BIA provided estimates of phase angle (PhA), total body water (TBW), extracellular (ECW) and intracellular water (ICW), fat-free mass (FFM), fat mass (FM), and body cell mass (BCM). 

### 2.5. Biochemical Assessments

At each visit (T0–T3), fasting venous blood samples were collected and analyzed according to the protocols of the hospital’s central laboratory. The biochemical assessments included liver enzymes such as alanine aminotransferase (ALT) and gamma-glutamyl transferase (GGT), in order to monitor hepatic health throughout the intervention. Glycemic control was evaluated by measuring fasting plasma glucose and glycated hemoglobin (HbA1c). A comprehensive lipid profile was also obtained, comprising triglycerides, total cholesterol, high-density lipoprotein (HDL), and low-density lipoprotein (LDL)—the latter calculated using the Friedewald formula [25]. Renal function was assessed through serum, creatinine and uric acid levels. Additionally, fasting insulin was measured for HOMA-IR calculation (fasting glycemia × fasting insulin)/405, with a reference range of 0.23 to 2.5 [26]. Homocysteine concentrations and PCR were also assessed to evaluate metabolic and inflammatory responses to the dietary intervention

### 2.6. Statistical Analysis

All study data and variables were entered into a database. Statistical analyses were performed using SPSS Version 25 for Windows (SPSS Inc., Chicago, IL, USA). Differences between groups were assessed using appropriate statistical tests, including Fisher’s exact test, Chi-square test, Student’s *t*-test, Mann–Whitney U test, or McNemar’s test. The normality of data distribution was evaluated using the Shapiro–Wilk test. Non-normally distributed variables were analyzed using non-parametric statistics. Continuous variables were presented as mean ± standard deviation (SD) if normally distributed, or as median [interquartile range (IQR)] if non-normally distributed. Categorical variables were expressed as frequencies and percentages. A binomial exact distribution model was used to estimate the 95% confidence intervals (95% CI) for proportions. Statistically significance was defined as *p* values less than 0.05.

## 3. Results

### 3.1. Patients Included

A total of 52 infertile women with overweight or obesity provided written informed consent to participate in the study. Baseline characteristics and reproductive characteristics of all enrolled participants are summarized in Table 2. The majority were classified with obesity (class I or II) and presented with infertility primarily due to ovulatory dysfunction (29%), male factor infertility (23%), or unexplained causes (23%). Among these, 40 women (77%) attended the baseline visit and initiated the VLCKD protocol. The baseline characteristics of the women who began the diet were similar to the total enrolled population (Supplementary Table S3).

**Figure 1:**
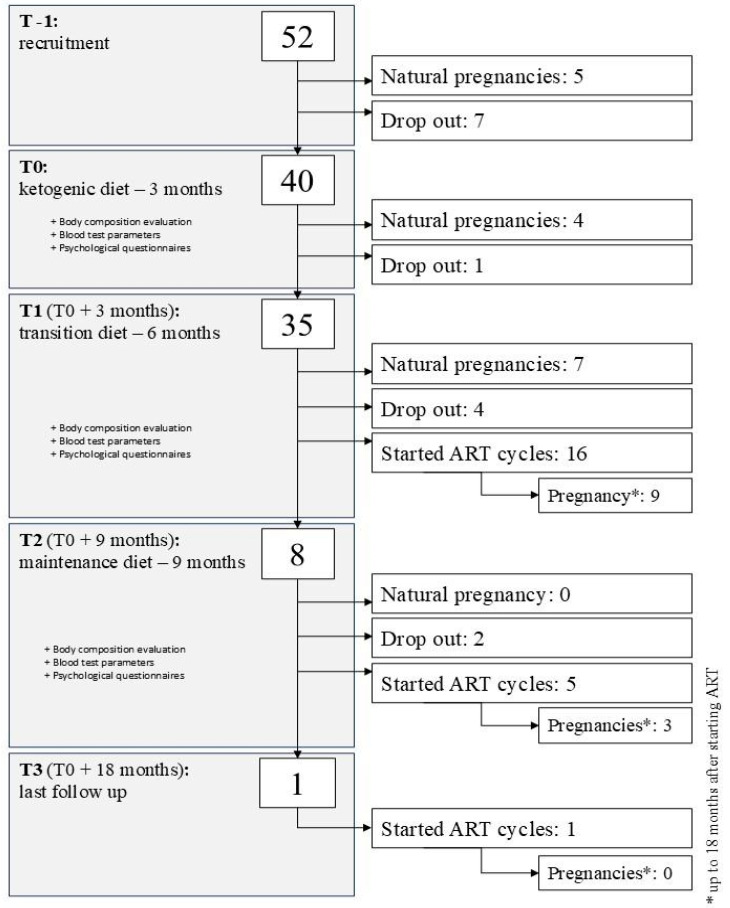
Visit and examination schedule and intervention flowchart. ART = Assisted Reproduction Therapy.

Five women (10%) conceived naturally before the dietary intervention could be initiated, while seven (14%) opted not to start the VLCKD. In total, twelve participants did not proceed to the baseline visit and were therefore excluded from secondary analyses since anthropometric, body composition, and biochemical assessments had not yet been conducted. The full flow of participants through the study phases is depicted in Figure 1.

### 3.2. Patients’ Follow-Up

Of the 40 women who began the VLCKD at baseline (T0), four were excluded from further analysis due to natural conception during the initial intervention period. Of the remaining 36 participants, 27 (75%) successfully completed the initial three-month VLCKD phase, achieving a reduction in body weight of more than 10% from baseline while preserving lean body mass—thus meeting the predefined criteria for intervention success. Six participants (15%) did not reach the ≥10% weight loss threshold by T1, two participants (6%) achieved the weight loss but failed to preserve lean body mass, and one participant dropped out. Considering all patients who initially agreed to participate, 51% (95% CI: 38–66%) (27/52) successfully completed the VLCKD intervention, achieving a ≥10% reduction in baseline weight while preserving lean body mass. This rate increased to 68% (95% CI: 51–81%) when considering only women who accepted the program (*n* = 40) and to 75% (95% CI: 58–88%) when excluding those who conceived early in the program, i.e., prior to completing the initial phase (*n* = 36).

Following the initial phase, 35 women entered the transition to maintenance (T1 phase). During this period, seven participants (20%) conceived naturally, and four (11%) discontinued participation and were lost to follow-up. Sixteen women (46%) proceeded to begin ART cycles, which resulted in nine pregnancies documented within the 18-month follow-up period.

Overall, eight women were non-adherent, of whom seven did not accept to enter the program after recruitment and one dropped out before ending the first phase. These eight women corresponded to 15% of the whole cohort (*n* = 52) and 19% of those who did not become pregnant before the end of the first phase (*n* = 43). 

Only eight participants returned for assessment at the end of the maintenance phase (T2). Of these, two dropped out before the final follow-up visit (T3). Five participants initiated ART cycles between T2 and T3, resulting in three additional pregnancies. Notably, all women who conceived during this final phase had achieved a weight reduction of more than 20% from baseline, with a mean weight loss of 21%.

One participant returned for the final follow-up, reporting a sustained weight loss of over 21%. She initiated ART shortly thereafter; however, no pregnancy was recorded during the 18-month follow-up period.

None of the participants discontinued the dietary regimen due to side effects or intolerance. However, some expected minor side effects such as halitosis, transient headache and constipation were reported. Participants experiencing constipation were managed with macrogol as needed.

### 3.3. Pregnancy and Neonatal Outcomes

A total of 11 women (27%) achieved a natural pregnancy after initiating the VLCKD. Among the remaining 29, 22 (76%) started an ART cycle in which 12 (55%) achieved pregnancy up to 18 months later. In total, 23 pregnancies occurred during the study period. Considering the analysis of the women who initiated the VLCKD (*n* = 40), the pregnancy success rate was 58% (95% CI: 42–71%). Pregnancy and neonatal outcomes are described in Table 3.

Expanding the analysis to the entire cohort of patients initially enrolled in the study (*n* = 52) and including the pregnancies that were achieved before starting the diet (*n* = 5), the overall pregnancy rate was 54% (95% CI: 41–67%). 

### 3.4. Results on Anthropometri Parameters and Body Composition

Changes in anthropometric and body composition measures between baseline (T0) and the end of the ketogenic phase (T1) are reported in Table 4. As expected, a significant reduction in BMI was observed. At recruitment, the median BMI was 36.6 kg/m^2^ [IQR: 33.4–39.9], which decreased to 30.8 kg/m^2^ [IQR: 27.3–33.6] following the three-month VLCKD phase. This was accompanied by a median reduction in WCirc of 12 cm [IQR: 10–16.5 cm].

Body composition analyses also revealed favorable changes: FM significantly decreased, while FFM percentage increased. Resistance (Rz), a marker linked to total body water, improved over time. Notably, despite the significant weight loss, reactance (Xc) and PhA, indicators of cell integrity and function, respectively, remained stable.

Additional body composition data were collected at T2 from the eight women who completed the transition phase. These results are detailed in Supplementary Table S3. The only participant who completed the full dietary program, including the maintenance phase, had a baseline BMI of 36.5 kg/m^2^. By the end of the ketogenic phase (T1), her BMI had decreased to 29.6 kg/m^2^ and remained relatively stable at 28.7 kg/m^2^ at the final follow-up visit (T3).

### 3.5. Blood Chemistry Assessments

The effects of the VLCKD on biochemical parameters between baseline (T0) and after three months of intervention (T1) are summarized in Table 5. Among the infertile women who completed the ketogenic phase, significant improvements were observed across multiple metabolic domains.

In terms of glucose metabolism, the VLCKD produced marked benefits. Fasting plasma glucose and insulin levels significantly decreased, accompanied by improvements in the homeostatic model assessment for insulin resistance (HOMA-IR) index, glycosylated hemoglobin (HbA1c), triglyceride-to-glucose ratio, and the triglyceride-glucose (TyG) indexes (all *p* < 0.001). 

Lipid profile parameters also improved significantly following the intervention. Reductions were observed in total cholesterol and LDL (*p* = 0.001), as well as in triglycerides (*p* < 0.001), while HDL levels increased (*p* = 0.004). 

Liver function markers showed favorable trends as well. Both ALT (*p* = 0.016) and GGT (*p* < 0.001) levels decreased significantly, suggesting a potential reduction in hepatic steatosis or liver stress associated with metabolic syndrome. Importantly, uric acid levels, often a concern during ketogenic diets due to increased catabolism, remained stable, with no significant change observed after the three-month period (*p* > 0.05).

CRP was used as a non-specific marker of inflammation and, despite decreasing after the first phase of the diet, did not reach statistical significance (*p* = 0.064).

## 4. Discussion

In this case-series study, we explored the feasibility and effects of a VLCKD in patients with overweight or obesity with infertility who were on the waiting list for ART. Our primary aim was to evaluate the proportion of participants who successfully followed the diet, defined as achieving at least 10% reduction in baseline body weight while preserving lean body mass during the initial three-month ketogenic phase. We also assessed the impact of the diet on anthropometric, body composition, and biochemical measurements. Notably, our findings showed a high success rate, with 75% of participants who initiated meeting this target and improvements observed across all key points evaluated. These results support the premise that the VLCKD is a highly effective and safe weight loss strategy for female patients with overweight or obesity in reproductive age and may represent a strategic intervention prior to ART initiation. On the other hand, it must be recognized that adherence was high but not optimal since some women (15–19%) did not accept to enter the program after recruitment or dropped out before ending the first phase.

Obesity is a well-established risk factor for subfertility, and numerous studies have demonstrated its negative impact on live birth rates and obstetric outcomes [4]. Unfortunately, maternal obesity is also associated with poorer IVF outcomes, including lower clinical pregnancy and live birth rates [6]. However, the effectiveness of preconception weight loss in reversing these effects remains under debate. While some studies suggest that a weight loss of at least 10% significantly improves ART outcomes [9,26], a recent meta-analysis found no benefit of pre-IVF weight loss in improving live birth rates among female patients with overweight or obesity [8]. It is worth noting that this meta-analysis excluded studies involving women with polycystic ovary syndrome (PCOS), a key condition contributing to obesity-related infertility. Additionally, the average weight loss achieved in the included studies was modest (mean loss ~5.5 kg), which may have limited the observed benefits. Supporting the importance of weight loss, a large cohort study involving real-world data from nearly 250,000 women found that weight reduction significantly improved pregnancy rate among those with both overweight or obesity and concomitant PCOS [27]. Reflecting this evidence, the European Association for the Study of Obesity now recommends a 5–10% weight loss over six months to improve fertility in infertile women with overweight or obesity [28].

The present study adds to this growing body of evidence supporting the effectiveness of the VLCKD by demonstrating that this dietary approach not only leads to significant short-term weight reduction but also promotes favorable changes in body composition and metabolic health in infertile patients with overweight or obesity in reproductive age. Consistent with previous reports, we observed substantial reductions in BMI and WCirc after just three months of intervention. Although follow-up data at six months post-intervention were available for only eight participants, nearly 60% of those who completed the VLCKD phase and did not become pregnant maintained their weight loss. Importantly, the weight reduction occurred without a significant loss of lean mass. In fact, our findings suggest preservation of fat-free mass, as evidenced by stable PhA and BCM/FFM ratio, along with an increase in BCM%, a marker of preserved metabolically active tissue [29].

An efficient diet to lose weight that is sustainable in the long term while preserving fat-free mass is particularly relevant given the risks associated with sarcopenic obesity, where fat loss occurs at the expense of muscle mass, potentially increasing cardiometabolic risk [30]. Therefore, weight loss strategies that promote fat mass reduction while preserving muscle mass and strength are ideal [17,19].

The improvements observed were not limited to anthropometric measures. Despite our patients with overweight or obesity exhibiting significantly altered metabolic profiles at recruitment, biochemical analyses revealed significant reductions in fasting glucose, insulin, HbA1c, lipid profile parameters, and liver enzymes. These findings suggest enhanced insulin sensitivity, improved lipid metabolism, and better hepatic function. Furthermore, glucose control, as shown by meta regression analysis, is associated with reduced risk of many pregnancy complications related to diabetes [31]. CRP, a nonspecific marker of inflammation, also improved during the first phase of the VLCKD, although the change did not reach statistical significance and values got closer to, but a little bit above, the reference range (0.3 mg/dL).

Regarding reproductive outcomes, a total of 23 pregnancies were achieved during the study: 11 natural and 12 following ART procedures. Among women who underwent ART after the beginning of dietary intervention, more than half achieved pregnancy, a rate markedly higher than the ~27% typically reported in female patients with overweight or obesity with PCOS [32]. While causality cannot be definitively established in this case-series design, these findings suggest a potential role for VLCKD in enhancing natural reproductive success in female patients with overweight or obesity, especially in a group composed by almost ¼ of women with disovulatory disorders. Even a modest weight loss of 5–10% of body weight can restore or improve menstrual cycles, resuming ovulation in anovulatory women, such as those affected by PCOS, significantly increasing the likelihood of conception [33]. The rate of natural pregnancies after the start of the VLCKD reached 50% (*n* = 7) among women with ovulatory disorders (*n* = 14), further supporting this potential benefit (Supplementary Table S3).

This study has important limitations. The absence of a control group limits the ability to infer causality or directly compare VLCKD with standard dietary or lifestyle interventions. Although case-series designs do not require controls, the lack of randomization introduces potential biases and prevents definitive conclusions about the superiority of the intervention. Additionally, the sample size, though sufficient for pilot observations, was relatively small, and follow-up rates decreased over time, reducing the generalizability of our findings and precluding a more detailed adjustment for potential confounders such as age, PCOS prevalence, or prior ART history. Moreover, although the Italian guidelines do not recommend routine monitoring of ketone levels as part of VLCKD management [23], such measurements could have provided additional information regarding dietary adherence. In our study, adherence was inferred indirectly through clinical indicators such as reduced hunger and weight loss, but ketone levels in blood or urine were not monitored. Lastly, the high success rate observed and the substantial proportion of women who achieved pregnancy, both naturally and ART-related, support the feasibility and potential utility of VLCKD in this population. These results should therefore be considered hypothesis-generating and underscore the need for larger, controlled, randomized studies to confirm the impact of preconception VLCKD interventions on fertility outcomes.

Among the strengths of this study is its real-world setting, which mirrors clinical practice where women have to experience significant wait times before ART initiation. This period may be underutilized, and our findings suggest it offers a unique opportunity for impactful dietary interventions such as VLCKD. The high and extremely rapid success observed also suggests that VLCKD is acceptable and well-tolerated, especially among women actively trying to conceive, representing a viable first-line approach for motivated women without contraindications to ketosis, offering both metabolic and reproductive benefits. Given the high global prevalence of obesity and its rising trend among reproductive-aged women, effective weight loss preconception interventions are needed. However, the effects of BMI on IVF outcomes as well as preconception weight loss still require further investigation.

## 5. Conclusions

This prospective observational case series study demonstrates that a structured VLCKD intervention is feasible, well-tolerated, and associated with significant improvements in weight, especially in body composition, metabolic health, and potentially fertility outcomes in female patients with overweight or obesity awaiting ART. While these findings are promising, they should be confirmed in larger, randomized controlled trials to better understand the magnitude of effect, elucidate underlying mechanisms, and assess long-term reproductive and obstetric outcomes.

## Figures and Tables

**Table 1:** Overview of the dietary intervention.

**Phase T0–T1** **(3 Months)**	**Phase T1–T2** **(6 Months)**	**Phase T2–T3** **(12 Months)**
<800 kcal/day 30–50 g/day of carbohydrate (13% of the total amount of kcal/day) 4 meal replacements per day plus low-glycemic-index vegetables (lunch and dinner) Standardized supplementation protocol (multivitamin complex and omega-3 fatty acids) Protein 35%, Fat 52% (12% saturated), CHO (carbohydrates) 13%, 12 g Fiber	1350 kcal/day >50 g/day of carbohydrate beginning with low-glycemic index options No meal replacements No Standardized supplementation protocol Protein 25%, Fat 43% (13% saturated), CHO 32%, 19 g Fiber	1450 kcal/day Mediterranean style pattern Protein 22%, Fat 30% (11% saturated), CHO 48%, 28 g Fiber

**Table 2:** Baseline characteristics of the whole cohort.

Characteristics	Whole Cohort (*n* = 52)
Age (years)	35 [30–39]
BMI (Kg/m^2^)	36.5 [32.6–39.8]
Duration of infertility (years)	2 [1–5]
FSH (IU/L)	6.3 [4.8–7.3]
AMH (IU/mL)	2.4 [1.6–3.8]
TSH	2.0 [1.5–2.3]
AFC	15 [7–20]
Previous Surgery	2 (4%)
Previous IVF	9 (17%)
Previous Pregnancies	15 (29%)
Previous live births	9 (17%)
Indication to IVF	
Ovulatory disorder	15 (29%)
Unexplained	12 (23%)
Male Factor	12 (23%)
Mixed	6 (11%)
Tubal Factor	4 (8%)
Genetic	2 (4%)
Endometriosis	1 (2%)

Data are reported as median [interquartile range] or number (percentage). Legend: AMH = Anti-Mullerian Hormone; AFC = Antral Follicle Count; BMI = Body Mass Index; IVF = in vitro fertilization; FSH = Follicle-Stimulating Hormone; TSH = Thyroid-Stimulating Hormone.

**Table 3:** Pregnancy and neonatal outcomes of women who started VLCKD.

Characteristics	Clinical Pregnancy (*n* = 23)
Lost to follow up	3 (13%)
Live birth	20/20 (100%)
Twin pregnancies	1/20 (5%)
Modes of delivery	
Vaginal	10/20 (50%)
Cesarean section	10/20 (50%)
Pregnancy complications with live birth	
Gestation Diabetes	6/20 (30%)
Hypertension	2/20 (10%)
Preterm labor (<37 w)	2/20 (10%)
Neonatal outcomes (*n* = 21)	
Sex	
Male	11 (52%)
Female	10 (48%)
Birth weight (g)	3150 [2915–3403]
Low birth weight (<2500 g)	3/21 (14%)

Data are reported as median [interquartile range] or number (percentage).

**Table 4:** Anthropometric parameters and body composition before and after a VLCKD.

Variables	Visits		
	T0	T1	*p*
Anthropometrics			
BMI (kg/m^2^)	36.6 [33.4–39.9]	30.8 [27.3–33.6]	<0.001
WCirc (cm)	105 [99–118]	93 [87–102]	<0.001
WCirc/H	0.66 [0.61–0.72]	0.58 [0.52–0.63]	<0.001
Body composition			
FFM (%)	56.8 [54.2–59.4]	63.0 [58.4–67.4]	<0.001
FM (%)	43.2 [39.9–45.8]	36.4 [32.5–40.8]	<0.001
FM/FFM	0.76 [0.67–0.81]	0.59 [0.49–0.69]	<0.001
BCM (%)	30.8 [27.1–32.6]	32.5 [29.9–35.4]	<0.001
BCMI	11.0 [10.4–12.4]	10.0 [9.5–11.4]	<0.001
BCM/FFM	0.54 [0.50–0.55]	0.5 [0.5–0.5]	0.065
Rz	467 [419–512]	492 [427–553]	<0.001
Xc	55 [50–63]	54 [49–61]	0.491
PhA	6.9 [6.0–7.2]	6.5 [5.9–6.9]	0.124

Data are reported as median [interquartile range] or number (percentage); Legend: BCM = Body Cell Mass; BCMI = Body Cell Mass Index; BMI = Body Mass Index; FFM = Fat Free Mass; FM = Fat Mass; H = Height; PhA = Phase angle; Rz = Resistance; Wcirc = waist circumference; Xc = Reactance.

**Table 5:** Glycemic measures and biochemical parameters.

Variables	Visits		
	T0	T1	*p*
Fasting glycemia (mg/dL)	93 [90–101]	85 [79–91]	<0.001
Fasting insulin (µU/mL)	23.1 [12.0–31.7]	10.1 [6.5–14.2]	<0.001
HOMA-IR	5.3 [2.7–7.2]	1.9 [1.2–3.3]	<0.001
HbA1c (%)	35 [32–39]	33 [30–34]	<0.001
TyG	5220 [3373–8057]	3476 [2829–4280]	<0.001
TyG LN	8.6 [8.1–9.0]	8.2 [7.9–8.4]	<0.001
Total cholesterol (mg/dL)	197 [174–227]	165 [150–191]	<0.001
HDL cholesterol (mg/dL)	54 [43–63]	46 [38–51]	0.004
LDL cholesterol (mg/dL)	121 [100–144]	103 [85–118]	0.001
Triglyceridemia (mg/dL)	110 [77–154]	81 [68–103]	<0.001
Total low-risk lipoprotein cholesterol (mg/dL)	21 [15–28]	17 [14–21]	0.001
ALT (ng/mL)	23 [16–37]	18 [15–24]	0.016
GGT (ng/mL)	18 [13–31]	10 [8–16]	<0.001
Uric acid (mg/dL)	4.9 [4.3–5.5]	5.5 [4.2–6.2]	0.157
C-reactive protein (mg/dL)	0.45 [0.19–0.84]	0.34 [0.11–0.68]	0.064

Data are reported as median [interquartile range] or number (percentage); Legend: ALT = Alanine aminotransferase; GGT = Gamma-Glutamyl Transferase; HbA1c = glycated hemoglobin; HDL = high-density lipoprotein; HOMA-IR = Homeostatic Model Assessment for insulin resistance; LDL = low-density lipoprotein; LN = Logarithmic normalization; TyG = Triglycerides-Glucose index.

## Data Availability

The original contributions presented in this study are included in the article/Supplementary Materials. Further inquiries can be directed to the corresponding author.

## Supplementary Materials

The following supporting information can be downloaded at https://www.mdpi.com/article/10.3390/nu17182930/s1: Supplementary Table S1. Composition of the ISOMED meal replacement (range of content per portion); Supplementary Table S2. Sample daily meal replacement plan with ISOMED VLCKD; Supplementary Table S3. Baseline characteristics, infertility factors, sequential measurements (T0–T3), and treatment outcomes in the study.

## Abbreviations

The following abbreviations are used in this manuscript:

ALT	Alanine Aminotransferase
AFC	Antral Follicle Count
AMH	Anti-Mullerian Hormone
ART	Assisted Reproductive Technologies
BCM	Body Cell Mass
BCMI	Body Cell Mass Index
BIA	Bioelectrical impedance analysis
BMI	Body Mass Index
ECW	Extracellular Water
FFM	Fat Free Mass
FM	Fat Mass
FSH	Follicle-Stimulating Hormone
GGT	Gamma-glutamyl transferase
H	Height
HbA1c	Glycated hemoglobin
HDL	High-density lipoprotein
HOMA-IR	Homeostatic model assessment for insulin resistance
ICW	Intracellular Water
IVF	In vitro fertilization
LDL	Low-density lipoprotein
LN	Logarithmic normalization
PCOS	Polycystic ovary syndrome
PhA	Phase Angle
Rz	Resistance
TBW	Total Body Water
TyG	Triglyceride/glucose
VLCKDs	Very low calorie ketogenic diets
WCirc	Waist circumference
Xc	Reactance
